# Influence of Relative Displacement on Surface Roughness in Longitudinal Turning of X37CrMoV5-1 Steel

**DOI:** 10.3390/ma14051317

**Published:** 2021-03-09

**Authors:** Michal Skrzyniarz, Lukasz Nowakowski, Edward Miko, Krzysztof Borkowski

**Affiliations:** 1 Department of Manufacturing Engineering and Metrology, Kielce University of Technology, al. Tysiaclecia Panstwa Polskiego 7, 25-314 Kielce, Poland; lukasn@tu.kielce.pl (L.N.); emiko@tu.kielce.pl (E.M.); 2 Department of Automation and Robotics, Kielce University of Technology, al. Tysiaclecia Panstwa Polskiego 7, 25-314 Kielce, Poland; kborkowski@tu.kielce.pl

**Keywords:** turning process, relative displacement, surface roughness, influencing factors, machining

## Abstract

The shaping process of surface texture is complicated and depends on many factors and phenomena accompanying them. This article presents the author’s test stand for the measurement of relative displacements in a tool–workpiece system during longitudinal turning. The aim of this study was to determine the influence of edge radius on the relative displacement between the tool and workpiece. The cutting process was carried out with inserts with different edge radii for X37CrMoV5-1 steel. As a result of the research, vibration charts of the tool–workpiece system were obtained. In the range of feed 0.03–0.18 mm/rev, the values of the standard deviation of relative displacements in the *x*-axis were obtained in the range of 0.36–0.78 μm for the insert with an edge radius of *r_n_* = 48.8 μm. As a result of the work, it was determined that for the feed value of 0.12 mm/rev for all inserts, the relative displacements are the smallest. As the final effect, the formula for forecasting the Ra roughness parameter was presented.

## 1. Introduction

The turning operation is widespread in manufacturing processes and is used in the industry to produce machine and equipment parts [1,2]. To reduce production costs, grinding is increasingly being replaced by turning. The task of the engineer designing the technological process of a workpiece is to select the proper machining parameters and their conditions to guarantee the required quality of the surface texture while maintaining the efficiency of the process. The surface texture has a significant impact on the functional and operational properties of the resulting workpieces [3,4]. The surface texture shaping process is complicated and depends on many variable factors [5,6]. In the lists created by the authors of previous works, the factors influencing shaped surfaces have been selected and divided into main groups related to [7,8] cutting tool, machining parameters, properties of the machined material, and phenomena accompanying the machining [9,10]. Phenomena involved in machining that affect the surface quality are: machining forces, chip forming and detachment, friction in the machining zone, vibration acceleration, displacement, built-up edge, and temperature distribution and flow [11]. In many works, models for predicting the roughness values of *Ra* and *Rt* are often found, as these are the most common industry requirements for roughness for manufactured parts. Most often, these models show a stereometric–kinematic representation of the corner radius on the machined surface. These models contain a geometric factor related to the corner radius, and the feed rate dominates as a kinematic factor. The most well-known formula for forecasting the Ra parameter was developed by Shaw and Crowell [12]. This formula was the basis for the development of many other formulas. In [13], it was indicated that the process of shaping the surface geometry also influences the relative displacements of the workpiece and tool, and researchers developed their formula with relative displacements. In [14], the researcher developed a formula for forecasting the arithmetic average value of surface roughness. In this formula, the most important factors were: feed rate, tool nose radius, relative displacements, and minimum uncut chip thickness phenomenon. Researchers stated that in their formulas that the cutting edge radius is included indirectly because it has an influence on the deviation of relative displacement, which is the main reason for the article.

A separate group of models for forecasting the surface roughness contains only factors related to the technological parameters of machining, i.e., feed rate, cutting speed, and depth of cut. These models mainly contain two to six factors affecting the roughness of the produced surfaces, and it is difficult to try to use them to predict surface roughness because this formula is dedicated to the exact tool, workpiece, and materials. Similar work was also carried out for milling processes, where researchers analysed the effect of displacement on the quality of the surfaces produced [15,16].

The aim of this study was to determine the influence of edge radius on the relative displacement between tool and workpiece and on the surface roughness. This publication presents the author’s test stand for measuring relative displacements in the tool–workpiece system during longitudinal turning, which is equipped with two eddy current sensors. The use of two sensors allows measuring displacements in two directions: radial and tangential simultaneously. The way of signal filtration is described, and the value of displacements recorded in two directions during turning is determined. The effect of the work is to determine the impact of feed rate and edge radius on the standard deviation of vibrations generated in the system and their impact on the surface roughness.

## 2. Materials and Methods

**Figure 1:**
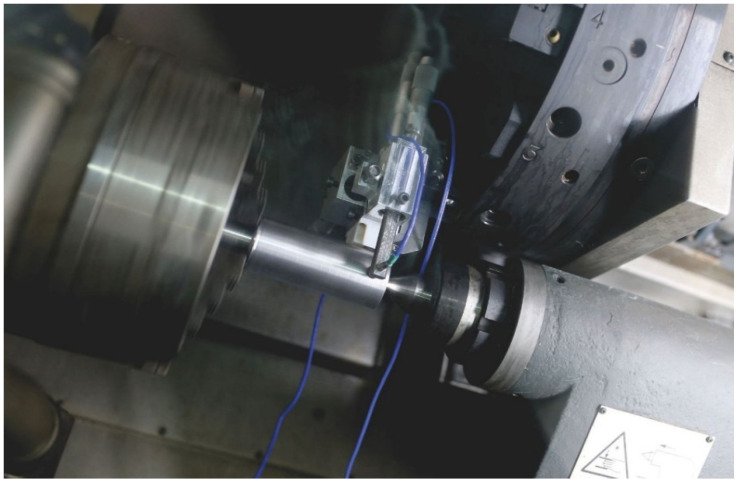
View of the measuring head mounted on the DMG Alpha 500 machining centre.

**Figure 2:**
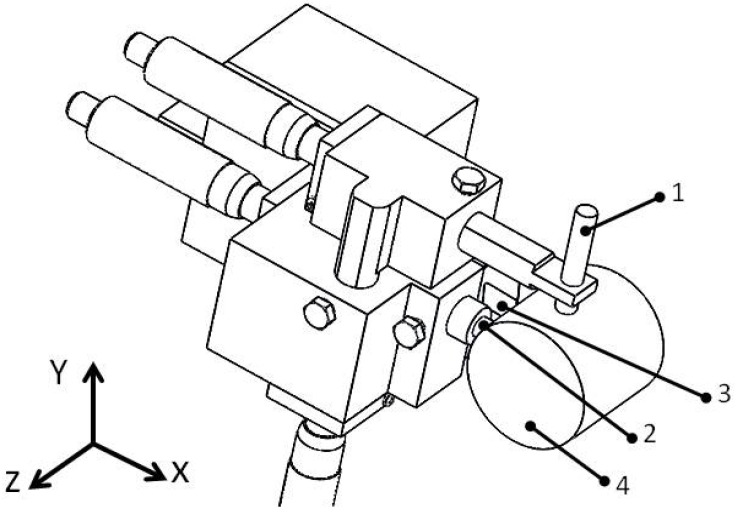
Scheme of the measuring head mounted on the lathe: (1) Eddy Current Sensor 1; (2) Eddy Current Sensor 2; (3) insert; (4) workpiece.

Generally, the test stands for measuring displacement in tool–workpiece systems are built mainly using three types of sensors: acceleration, inductive, and optical. Each method of measuring the displacement using any of the above-mentioned sensors has its limitations [17]. Two eddy current sensors have been used to build the stand. The selected (EPU 1, Micro-Epsilon, Ortenburg, Germany) sensor has a large linearity range, a repeatability error of less than 1 µm, a data collection frequency from the current output of up to 25 kHz, a resolution of 0.05 µm, and temperature impact compensation. Before the tests, the sensors were calibrated by a laser interferometer (XL-80, Renishaw, New Mills, UK). As a result of the work performed, a head for measuring relative displacements was built which is illustrated in Figure 1. Figure 2 shows the scheme of the measuring head.

The aim of the tests was to determine the impact of such parameters as machining speed (*v_c_*), feed rate (*f*), and edge radius (*r_n_*) on the value of generated movements of the tool in relation to the workpiece during longitudinal turning and on surface roughness parameters. The depth of cut (*a_p_*) was fixed at 0.35 mm. Experimental tests were carried out without the coolant for different machining parameters and inserts with different coatings.

The prepared material in the form of a cylinder was previously machined in the same clamping with a feed rate *f* = 0.06 mm/rev and a machining speed *v_c_* = 150 m/min. This operation was conducted to avoid errors associated with changing the sample fixture so that once defined, the sample fixture system does not change during the machining tests. Machining parameters related to surface preparation were selected so that the surface produced would not introduce errors for reading the displacements by the sensors through high values of surface height parameters. The preliminary preparation of samples, e.g., on a grinder, was deliberately abandoned. This operation could allow obtaining a smooth surface without such large dimensional errors as those we get from turning. Material grinding could introduce surface magnetisation of the material, which would have a negative effect on position reading by the eddy current sensors.

The CoroTurn 107 SDJCL 2020K 11 (Sandvik, Stockholm, Sweden) tool was used for all turning operations. The characteristics of the used tools are specified in Table 1. The criterion for the selection of inserts was the variable edge radius (*r_n_*). Due to the lack of information about the edge radius in the catalogues of cutting tool manufacturers, it was decided to choose inserts of different thickness and type of coatings so as to vary the *r_n_* parameter as much as possible. It was decided to choose three types of cutting inserts: DCMT 11 T3 08—PF 4325, DCMT 11 T3 08—MF 1105, and DCGT 11 T3 08—UM 1125.

After selecting the cutting inserts, the edge radius (*r_n_*) was measured. By selecting the inserts with different types and technologies of coating, the difference in edge radius was obtained. For 4325 grade inserts, the edge radius *r_n_* is in the range of 44.4–55.9 µm; for the 1105 grade, the radius varied between 29.6 and 38.9 µm. The smallest edge radius values were measured for the 1125 grade inserts from 5.3 to 11.5 µm. All cutting conditions used for the turning operations are specified in Table 2.

Before starting the machining process, it was decided to check the accuracy of the corner radius *r_ε_* with (O-INSPECTO, ZEISS, Jena, Germany) multisensor measuring machine. From the measurements of the corner radius involved in the experiment, it was found that they are within a manufacturing tolerance of *r_ε_* = 800 ± 12 μm.

X37CrMoV5-1 steel was selected for the machining tests. Its chemical composition is presented in Table 3. This steel was selected because of the dynamic chip detachment during machining. Before the machining tests, the hardness of the steel was measured using a Rockwell hardness tester. The tested material hardness was 34.9 ± 5 HRC. Thirty hardness measurements were made on a Rockwell hardness tester using a 120° diamond cone. All material samples were produced from a single supplied piece of bar, so measurements were made on randomly selected samples. The semifinished product for the machining tests was an around-rolled, annealed rod with a diameter of Ø 50.8 mm made of X37CrMoV5-1 steel. All the machining tests were carried out on a 4-axis DMG ALPHA 500 machining centre (DMG, Germany).

## 3. Results and Discussions

On the basis of the obtained values of relative displacements during turning, the standard deviation and the peak-to-trough amplitude of relative displacements in the tool–workpiece system were determined.

**Figure 3:**
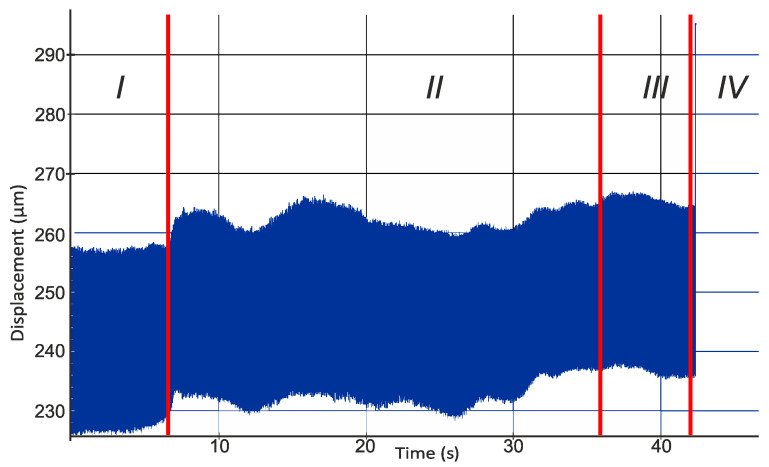
Displacement diagram of the tool relative to the workpiece from the sensor mounted in the direction of the *x*-axis for the machining conditions: *v_c_* = 360 m/min; *f* = 0.03 mm/rev; *a_p_* = 0.35 mm; *r_n_* = 48.6 µm; *n* = 2460 rpm.

Figure 3 shows an example of a recorded signal from an eddy current sensor measuring relative displacements in the direction of the *x*-axis on the machining centre. The parameters with which the machining was carried out for this example are: cutting speed *v_c_* = 360 m/min, feed rate *f* = 0.03 mm/rev, and depth of cut *a_p_* = 0.35 mm. In addition, the machining was carried out with a DCMT 11 T3 08 PF 4325 insert with an edge radius *r_n_* = 48.6 µm.

Figure 3 shows a registered displacement signal between the tool and the workpiece from which four characteristic zones can be distinguished. In the first zone, the tool with the attached sensors is moved to the starting position. Due to the fact that the data-collecting sensor is located in front of the tool, displacement is measured before the tool enters the material. This is due to the activated machine spindle rotation. Then the displacement diagram shows a characteristic change in its stability in about 6 s. This change is caused by the machining start and the kinematic system’s unbalance from the set balance. In this case, an increase in the values recorded by the sensor was observed. This change is caused by pushing the workpiece away from the tool due to the distribution of forces during machining. This phenomenon is followed by a turning process lasting up to about 38 s. In this respect, it was observed that the process is not stabilised and that the movements occurring within it do not have an established course. The graph between 38 and 42 s is similar to the one before machining. This is due to the temporary stop of the tool over the rotating workpiece after reaching the final position. The entire machining process was carried out at a length of 40 mm. In the final phase, there is a vertical line connected with the tool’s track out to the tool replacement point defined in the control program, which causes the sensor to move outside its measuring range.

**Figure 4:**
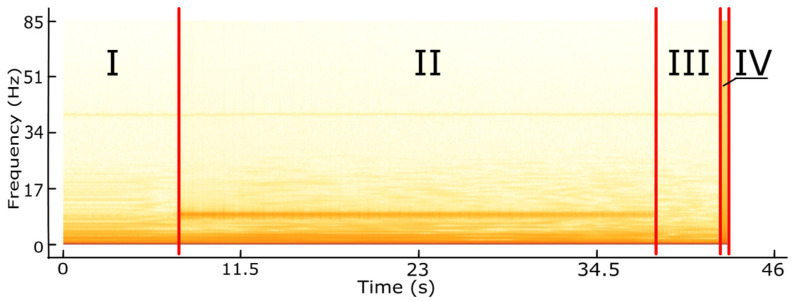
Amplitude spectrum spectrogram for each t moment of the recorded displacement signal based on the graph in Figure 3.

The spectrogram for the recorded displacement signal is shown in Figure 4. The spectrogram represents the amplitude spectrum of the signal for each moment t for which a measurement is recorded. Its design consists of determining amplitude harmonics for each component. A spectrogram is a temporary Fourier transform of the signal, displacements in this case. By analysing the spectrogram image, four characteristic zones resulting from the experiment can also be found in its construction. The first zone, where there are no visible characteristic changes in the spectrogram, registers the tool’s approach to the workpiece and its halt at its starting position before machining starts. A characteristic horizontal line is visible along the entire length of the graph in the middle of its height, which registers the frequency of the rotation of the machine spindle during the machining process.

**Figure 5:**
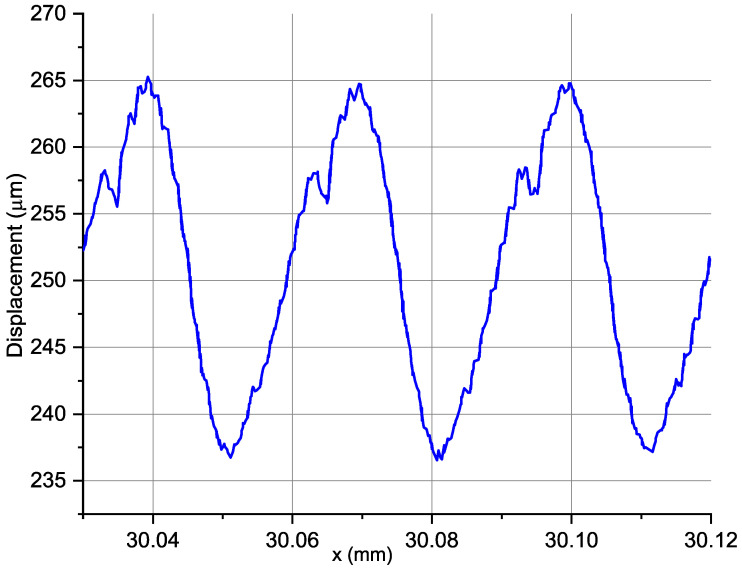
Example of signal slice after trend removal: *vc* = 360 m/min, *f* = 0.03 mm/rev; *ap* = 0.35 mm; *r_n_* = 48.6 µm; *n* = 2460 rpm.

Due to the fact that the workpiece was clamped in a three-jaw self-centring chuck and supported by the tailstock centre, the system was the most stable at its beginning and end, while the centre of the workpiece had the greatest possibility of deflection due to forces during machining. A sample chart after trend removal is shown in Figure 5. This graph shows only three adjacent full rotations of the workpiece due to the legibility of the presented content.

**Figure 6:**
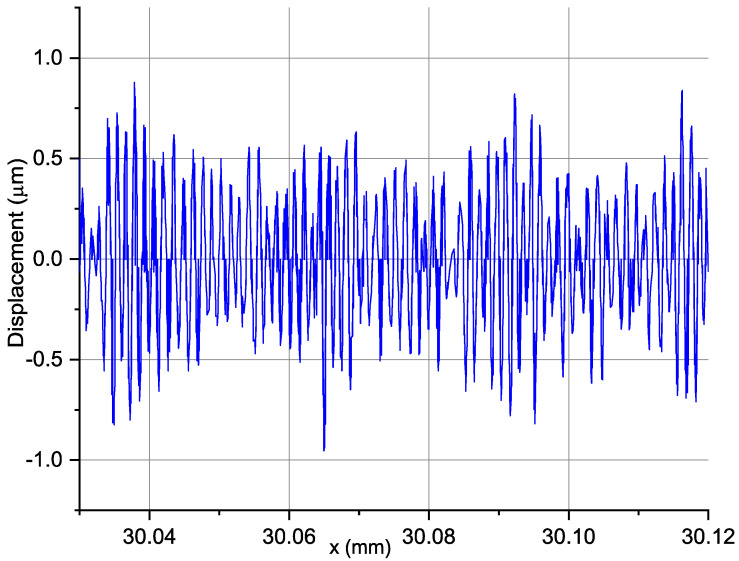
The signal in the range from 15 to 150 wavelengths per revolution: *v_c_* = 360 m/min; *f* = 0.03 mm/rev; *a_p_* = 0.35 mm; *r_n_* = 48.6 µm; *n* = 2460 rpm.

After removing the trend, the next step of the signal analysis was to remove the constant component, i.e., the average profile value, and the first harmonic component responsible for the noncentricity and shape errors were also removed. Figure 6 shows the signal based on which standard deviation parameters of displacements, their variance, and amplitude of displacements will be calculated. The value of the peak-to-trough amplitude for a given section is 1.88 µm.

**Figure 7:**
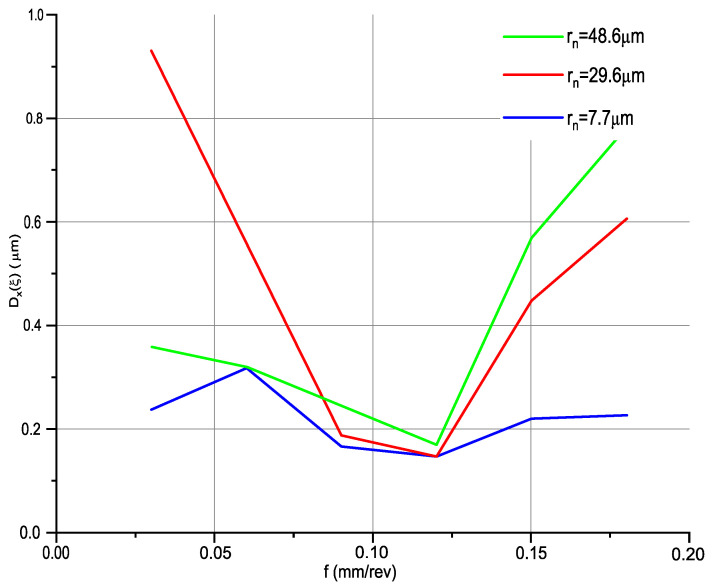
Impact of the feed rate and edge radius on the standard deviation of vibrations generated in the *x*-axis direction: *v_c_* = 360 m/min; *f* = 0.03−0.21 mm/rev; *a_p_* = 0.35 mm.

After preparation of the signal filtration procedure to obtain displacements occurring during the longitudinal turning process, calculations of the standard deviation of relative displacements D(ξ) were performed for individual samples. Figure 7 shows the impact of feed rate and edge radius on the displacements generated in the tool–workpiece system. The test range was from 0.03 to 0.18 mm/rev, while the edge radius occurred in three configurations: 48.6, 29.6, and 7.7 µm.

Analysing Figure 7, it was noted that the displacement values for all cutting inserts are similar. The displacement values initially decrease from a feed rate of 0.03 mm/rev and reach their minimum for a feed rate of 0.12 mm/rev. All minimum values were recorded for each type of insert. From a feed rate of 0.12 mm/rev, displacement increases. This graph shows that the optimum machining condition due to the value of generated displacement in the examined system for the radius of all edges is 0.12 mm/rev. This is due to the fact that the machining process becomes more stable due to better chip shaping and detachment.

For a feed rate of 0.03 mm/rev, displacements are largest for DCMT 11 T3—MF 1105, more than double that of the others for the same feed rate. For feed values of 0.15 and 0.18 mm/rev, the differences between the displacement values are significant; this is due to the increase in the chip cross-section, which increases the cutting resistance.

After the machining tests and simultaneous measurement of relative displacements during machining, the next part of the experiment is based on 3D surface topography measurement. The optical profilometer was used to measure surface topography: Talysurf CCI Lite (Taylor Hobson, Great Britain). Apart from the analysis of 3D parameters of the surface profile, a 2D profile analysis was also carried out and statistics for 2D measurements were developed based on 103 out of 1024 registered profiles. Isometric images obtained to extract the roughness were subjected to Gaussian filtering with a 0.8 filter.

**Figure 8:**
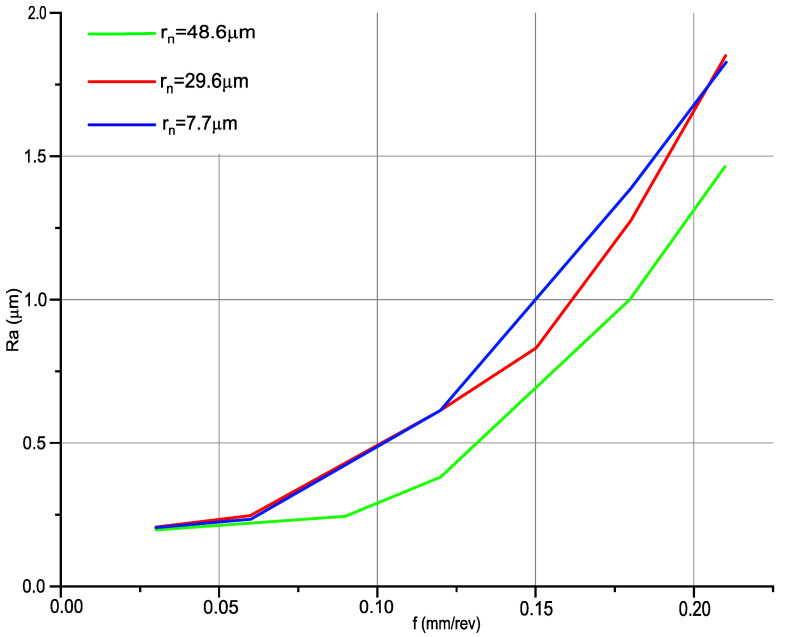
Effect of feed rate and edge radius on the surface roughness parameter *Ra*: *v_c_* = 360 m/min; *f* = 0.03–0.21 mm/rev; *a_p_* = 0.35 mm.

When analysing the effect of feed rate on the 2D and 3D parameters of surface roughness, it was noticed that all parameters behave in the same way. As the feed rate increases, the roughness parameters increase due to the stereometric–kinematic mapping of the cutting edge on the material surface. The differences between the roughness parameters are low, which is related to the different edge radiuses used in the experiment. Figure 8 shows the effect of feed rate and edge radius on the surface roughness parameter *Ra*.

For feed rates of 0.03 and 0.06 mm/rev, it was noticed that the difference between the Ra parameter for the same technological parameters of machining is small. This difference is noticed for feed rates above 0.09 mm/rev. The smallest values of roughness parameter Ra were obtained for machining with an edge radius of 48.6 µm, while the highest values were recorded for the smallest edge radius of 7.7 µm. The difference in roughness between machining with a 29.6 to 7.7 µm edge radius is small. The differences in the Ra parameter increase with the feed rate; the highest value was recorded at a feed rate of 0.21 mm/rev and is 0.364 µm.

**Figure 9:**
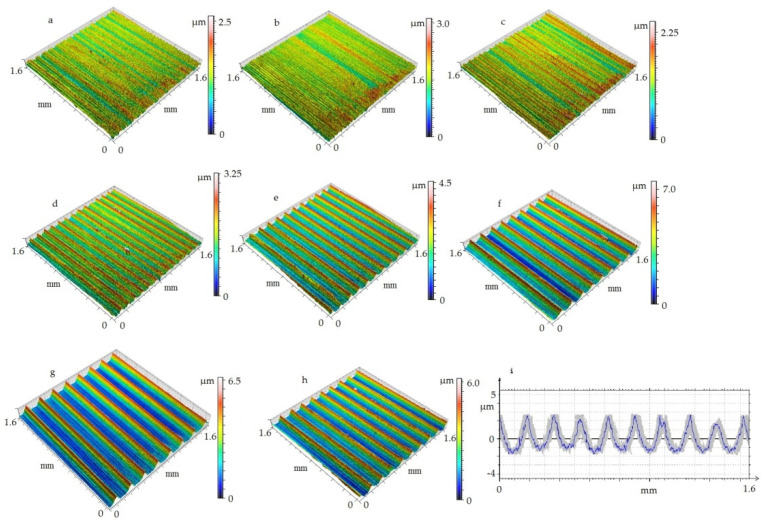
Examples of 3D and 2D surface topography, *v_c_* = 360 m/min, *a_p_* = 0.35: (**a**) *f* = 0.03 mm/rev; (**b**) *f* = 0.06 mm/rev; (**c**) *f* = 0.09 mm/rev; (**d**) *f* = 0.12 mm/rev; (**e**) *f* = 0.15 mm/rev; (**f**) *f* = 0.18 mm/rev; (**g**) *f* = 0.18 mm/rev; *r_n_* =29.6 μm; (**h**) *f* = 0.18 mm/rev; *r_n_* = 7.7 μm; (**i**) *f* = 0.18 mm/rev; *r_n_* = 48.6 μm.

Figure 9 shows selected examples of 3D and 2D geometries. In the feed rate range of 0.03 to 0.09, there are few changes in the isometric images. This is due to the geometry of the cutting insert which has a tool nose radius of 0.8 mm. Beyond a feed rate of 0.12 mm/rev, increasing changes in the isometric images are seen. This is due to the kinematics of the process, which resembles the cutting of micro threads. Table 4 shows the data that was used to develop the predictive model for the surface roughness parameter Ra taking into account the displacements between the tool and the workpiece. The *Dx(ξ)* and *Dy(ξ)* parameters were determined on the basis of the obtained results of relative displacement tests of the tool and workpiece using the constructed test stand.

The model for predicting the surface roughness Ra parameter including relative displacements in the tool–workpiece system takes the form:

(1) $$Ra=\sqrt{\frac{k_{D\left(\xi \right)}\cdot f^{4}}{972r_{\varepsilon }^{2}}+\frac{D_{c}{\left(\xi \right)}^{2}}{4}}$$

In this model, *D_c_(ξ)* represents the displacement variance for a given feed rate based on displacements in the direction of the machine’s *x*- and *y*-axes.

(2) $$D_{c}\left(\xi \right)=\sqrt{D_{x}{\left(\xi \right)}^{2}+\;D_{y}{\left(\xi \right)}^{2}}\;$$

The formula additionally takes into account the coefficient describing the average value of displacements in both axes of the cutting tool *k_D_*_(*ξ*)_.

(3) $$k_{D\left(\xi \right)}=\bar{D_{c}\left(\xi \right)}\;$$

In addition to the impact of such parameters as feed rate, corner radius, and relative displacements in the tool–workpiece system, the model also indirectly takes into account the edge radius by its impact on the value of generated displacements during machining.

Based on the measurement data and model (1) for predicting the roughness parameter Ra in Table 5, the results of roughness measurements of produced surfaces (*Ra_d_*) were compared with the data predicted by the developed formula (*Ra_t_*).

**Figure 10:**
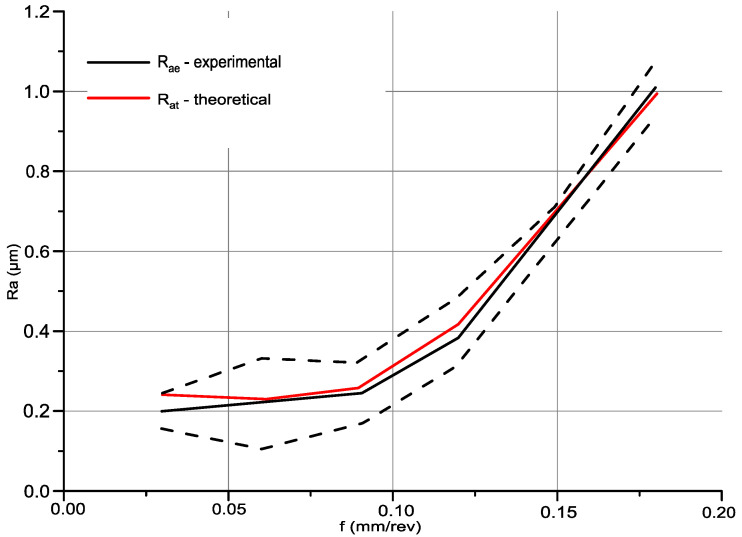
A graph showing the measured and projected Ra parameter of the longitudinally turned surface.

Figure 10 shows a graphical verification of the model together with the results of measurements of the roughness Ra parameter. The red line marks the predicted value of parameter Ra using the developed Equation (1). The solid black line describes the roughness results obtained for longitudinal turning, with a constant machining speed *v_c_* = 360 m/min. The dashed black lines are used to determine the mistrust intervals *±2σ* based on 103 selected sections from 1024 recorded during measurement on the Talysurf CCI optical profiler.

The analysis of Figure 10 together with the data contained in Table 5 showed that the model for predicting the roughness Ra parameter in the feed rate range *f* = 0.03–0.18 mm/rev is within the confidence interval determined for the measured values. Pearson’s correlation coefficient between measured and predicted values is 0.99, which indicates a strong correlation between the variables studied.

## 4. Conclusions

The aim of this work was to determine the influence of edge radius on the relative displacement between tool and workpiece. As a result of the work, a test stand for relative displacements in the tool–workpiece system was designed and constructed using two eddy current sensors. The ranges of the machine tool’s stable operation were also determined from the system vibration characteristics. To sum up, the most important conclusions from the tests described above are as follows:

1. Surface roughness during continuous machining is shaped by a number of factors, the most important of which are: the stereometric and kinematic mapping of the cutting edge and relative displacements in the tool–workpiece system. The roughness parameter Ra was changed for all inserts in the range from 0.205 to 1.85 μm. The smallest values of the roughness Ra parameter were obtained for the DCMT 11 T3 08—PF 4325 insert.
2. The standard deviation of the vibration for all inserts is in the tested range from 0.15 to 0.93 μm. The highest values were obtained for the DCGT 11 T3 08—UM 1125 insert. The range of stable machine tool operation due to the value of generated relative displacements in the tool–workpiece system for inserts with a corner radius *r_ε_* = 0.8 mm is 0.09–0.12 mm/rev. When the feed rate exceeds 0.12 mm/rev, the standard deviation of vibration increases.
3. The developed experimental model is used to forecast the parameter Ra of longitudinally turned surface roughness. The factors involved in the shaping process of the roughness in the formula were: feed rate, corner radius, the standard deviation of the relative displacement in the tool–workpiece system, and indirect edge radius.

## Figures and Tables

**Table 1:** Parameters of tools.

Clearance Angle, *α*, °	Tool Cutting Edge Angle, *κ_r_*, °	Angle of the Tip, °	Cutter Overhang, mm	Cutting Edge Length, mm
7	93	55	40	11

**Table 2:** Specifications of cutting conditions.

Depth of Cut, *d*, mm	Cutting Speed, *v_c_*, m/min	Feed per Revolution, *f*, mm/rev
0.35	360	0.03, 0.06, 0.09, 0.12, 0.15, 0.18, 0.21

**Table 3:** Chemical composition of X37CrMoV5-1 steel.

Chemical Composition in wt. %									
Workpiece Material	Carbon, C	Silicon, Si	Manganese, Mn	Phosphorus, P	Sulphur, S	Chromium, Cr	Molybdenum, Mo	Vanadium, V	Iron, Fe
Max	0.41	1.2	0.5	up to 0.03	up to 0.02	5.5	1.5	0.5	≈90
Min	0.33	0.8	0.25	-	-	4.8	1.1	0.3	

**Table 4:** Data used to develop an experimental model to predict the roughness parameter *Ra*.

*f,* mm/rev	*r_ε_,* mm	*r_n_,* µm	*D_x_(ξ)*	*D_y_(ξ)*
0.03	0.8	48.6	0.36	0.33
0.06	0.8	48.6	0.32	0.28
0.09	0.8	48.6	0.26	0.17
0.12	0.8	48.6	0.18	0.17
0.15	0.8	48.6	0.57	0.28
0.18	0.8	48.6	0.78	0.28

**Table 5:** Comparison of the roughness parameter prediction model with actual surface parameters.

*f,* mm/rev	*Ra_d_,* µm	*Ra_t_,* µm	*σ,* µm
0.03	0.205	0.245	0.02
0.06	0.222	0.235	0.006
0.09	0.247	0.267	0.01
0.12	0.384	0.419	0.018
0.15	0.682	0.703	0.011
0.18	1.011	0.994	0.008
-	Correlation coefficient $R^{2}=0.99$		-

*f*—feed rate (mm/rev), *Ra_d_*—the experimental arithmetic average value of surface roughness (μm), *Ra_t_*—the theoretical arithmetic average value of surface roughness (μm), *σ*—standard deviation of Ra_t_ parameter (μm).

## Data Availability

The data presented in this study are available on request from the corresponding author.

## Nomenclature

*v_c_*	cutting speed (m/min)
*f*	feed rate (mm/rev)
*a_p_*	depth of cut (mm)
*r_n_*	cutting edge radius (μm)
*r_ε_*	tool nose radius (mm)
*n*	spindle speed (rpm)
*Ra*	the arithmetic average value of surface roughness (μm)
*Ra_t_*	the theoretical arithmetic average value of surface roughness (μm)
*Ra_t_*	the experimental arithmetic average value of surface roughness (μm)
*Dx*(ξ)	the standard deviation of relative displacement tool and workpiece in the X direction
*Dy*(ξ)	the standard deviation of relative displacement tool and workpiece in the Y direction
*Dc*(ξ)^2^	the variance of relative displacements
*k_D_*(ξ)	coefficient
